# Meta-analysis of the efficacy of probiotics to treat diarrhea

**DOI:** 10.1097/MD.0000000000030880

**Published:** 2022-09-23

**Authors:** Fujie Wang, Ting Zhao, Weiwei Wang, Qianqian Dai, Xianghua Ma

**Affiliations:** a Nutritional Department, The First Affiliated Hospital of Nanjing Medical University, Nanjing, China; b Department of Critical Care Medicine, the First Affiliated Hospital of Nanjing Medical University, Nanjing, China; c Nutritional Department, Xuzhou Cancer Hospital, Xuzhou China.

**Keywords:** diarrhea, irritable bowel syndrome, probiotics

## Abstract

To collect the published trials of probiotics in the treatment of diarrhea and to strictly evaluate and systematically analyze the efficacy of probiotics use for the prevention and treatment of patients with diarrhea.

**Methods:**

We searched domestic and foreign literature published between January 2016 and July 2022 to find randomized control trials that used probiotics to treat diarrhea. Only studies published in English were considered. The quality of the included literatures was assessed by using the methods provided in the Cochrane Handbook. Valid data were extracted and analyzed by meta- analysis using the Software RevMan5.2.

**Results:**

Total 16 trials and 1585 patients were included. The results of the meta- analysis showed that in comparison with the simple Western medicine treatment group or placebo, the added use of probiotics could improve stool frequency, stool morphology, and related irritable bowel syndrome symptoms.

**Conclusion:**

The added use of probiotics can further improve clinical outcomes in the patients with diarrhea; however, the implementation of larger and higher quality clinical trials is necessary to verify this conclusion.

## 1. Introduction

It’s reported that probiotics may supplement the intestinal microbiota and improve its microbiota characteristics.^[1]^ The effects of probiotics include improvement of mucosal barrier function, promotion of visceral hypersensitivity, effects on gastrointestinal motility, and regulation of immune responses.^[2–5]^ A number of different probiotic strains have been reported to improve various gastrointestinal illnesses, especially, diarrhea and other associated symptoms.

Probiotics offer promising therapeutic solutions for various health conditions, including gastrointestinal illnesses such as diarrhea, ulcerative colitis, and Crohn disease, etc. Microbial dysbiosis, which is an imbalance in the gastrointestinal microbiome, is a cause for the prognosis of such gastrointestinal conditions.^[6]^ Bacteriotherapy with probiotics helps to reprogram the microbial balance of the gut and restore healthy.^[7]^ Clinical efficacies of probiotics are strain dependent, and therapeutic effectiveness depends on specific clinical circumstances such as digestive and nondigestive disorders.^[8,9]^

Moderate-quality evidence suggests that probiotic prophylaxis results in a large reduction in *Clostridium difficile*-associated diarrhea without an increase in clinically important adverse events.^[10]^ Besides, the pooled evidence suggests that probiotics are associated with a reduction in antibiotic-associated diarrhea.^[11]^

Irritable bowel syndrome (IBS) is one of the most common gastrointestinal disorders, characterized by abdominal pain or discomfort associated with defecation and changes in bowel habits.^[12]^ The Rome III criteria defines IBS as recurrent abdominal pain or discomfort at least 3 days per month in the last 3 months (symptom onset at least 6 months prior to diagnosis) associated with 2 or more of the following: improvement with defecation, onset associated with a change in frequency of stool, or onset associated with a change in form or appearance of stool.^[13]^ IBS is classified into four subtypes based on the symptoms and diarrhea-predominant irritable bowel syndrome is more prevalent.^[14]^ An increase of Firmicutes-associated taxa, a depletion of bacteroidetes taxa and a significantly lower biodiversity of microbes happen in the intestinal microbiota of IBS patient.^[15,16]^ Thus, improving the composition of the intestinal microbiota has becomes to the target of IBS treatment.

Recent studies have shown that a disturbed gut microbiota may promote the development and maintenance of IBS.^[17–19]^ Alterations in the intestinal microbiota can contribute to IBS by increasing gut permeability, activating the mucosal inflammatory immune response, increasing visceral sensitivity, and altering intestinal motility.^[20–22]^ In addition, the onset of IBS following infective gastroenteritis and the involvement of small bowel bacterial overgrowth suggest that gut microbes play a role in IBS pathogenesis.^[23]^ IBS can also be seen as a “stress disease”, and there is evidence that the microbiome-gut-brain axis is disturbed in IBS.

Over the years, probiotics have been extensively studied, and several beneficial effects have been discovered, such as protection against colonization by pathogenic bacteria, regulation of the immune system, and enhancement of intestinal barrier function.^[24,25]^ In the recent years, the use of probiotics as a treatment for various types of severe diarrhea has been increasing.^[26,27]^

In this study, we searched for randomized controlled trials that used probiotics to treat diarrhea. Probiotics are the latest innovative and developed modern objects for the treatment of diseases and are highly praised by clinicians and patients. Research on its mechanism and efficacy has increased in recent years. The purpose of this study was to collect the published randomized controlled trials of probiotics in the treatment of diarrhea and to strictly evaluate and systematically analyze them, in order to provide a basis for the clinical application of probiotics.

## 2. Materials and Methods

### 2.1. Information sources and search strategy

The papers to be included in the meta-analysis were searched in the The National Library of Medicine, Excerpt Medica Database, Scopus, Clinicaltrials.gov, Web of Science, and Cochrane Library databases in July 2022. The search terms used were: “Probiotics” and “Diarrhea” as the subject words. Search relevant magazines and track and consult the relevant contributions in the reference contributions to avoid any loss. A manual search of possible references of interest was also performed. Only studies published in English were considered. We did not register the reviews of the authors of these articles. There exists no review protocol.

### 2.2. Inclusion/exclusion criteria for study selection

All human-associated studies were included if they met the following criteria: randomized Controlled Trial; had been diagnosed with diarrhea; were older than 18 years; and sufficient data of clinical outcome data. All the studies were excluded if they met the following criteria: animal experiment, review, mechanism research, case report, collection of papers, literatures of the incomplete data and duplicates, and not obtaining full manuscripts. Participants were excluded if they: were pregnant or lactating; suffered from mental illness; used anti-depressants, anxiety, or neurological or psychiatric medication.

The World Health Organization defines diarrhea as excretion 3 or more times a day without fecal shape, when the condition lasts for more than 2 days.^[12]^

### 2.3. Intervention

Controlled group: All participants received a placebo or single in accordance with diarrhea Treatment Guidelines Western medicine treatment, including low in fermentable oligosaccharides, disaccharides, monosaccharides, fibers, and polyols and antibiotic interventions; treatment group: on the basis of the controlled group, different kinds of probiotics were added.

The ethics of the study was approved by the ethics committee of the First Affiliated Hospital of Nanjing Medical University.

### 2.4. Outcome measures

The outcome will be the changes of index, which explicitly reported at least one of the following: abdominal pain, bloating, stool frequency, Bristol stool scale (BSS), Irritable Bowel Syndrome-quality of life score, fecal microbiota analysis, irritable bowel syndrome-severity scoring system score, irritable bowel syndrome-global improvement scale score, gastrointestinal symptom rating scale; Interleukin-10, Interleukin-6 and Interleukin-12.

### 2.5. Quality and bias assessment

For the retrieved literature, excluding the trials that did not meet the inclusion criteria, the quality of each contribution was evaluated according to the quality evaluation criteria of the Cochrane Handbook randomized controlled trial (www.cochrane-handbook.org).

### 2.6. Statistical analysis

RevMan5.2 software was used for statistical analysis. The counting data were weighed by odds ratio, and the measurement data were weighed by a weighted mean difference (WMD), both expressed under a 95% confidence interval (95% CI). Heterogeneity was analyzed by χ^2^ test when *P* > .05, or *I*^2^ < 50%. There was no statistical heterogeneity among the studies, and Fixed Effects Mode1 was used for meta-analysis. When *P* < .05 or *I*^2^ > 50%, it indicates obvious heterogeneity among studies, and the causes of heterogeneity should be analyzed. After removing studies with greater heterogeneity, the fixed-effects model was used for analysis. If the causes of heterogeneity cannot be found, a random effects model or descriptive analysis should be performed.

## 3. Results

### 3.1. Literature search

**Figure 1.**
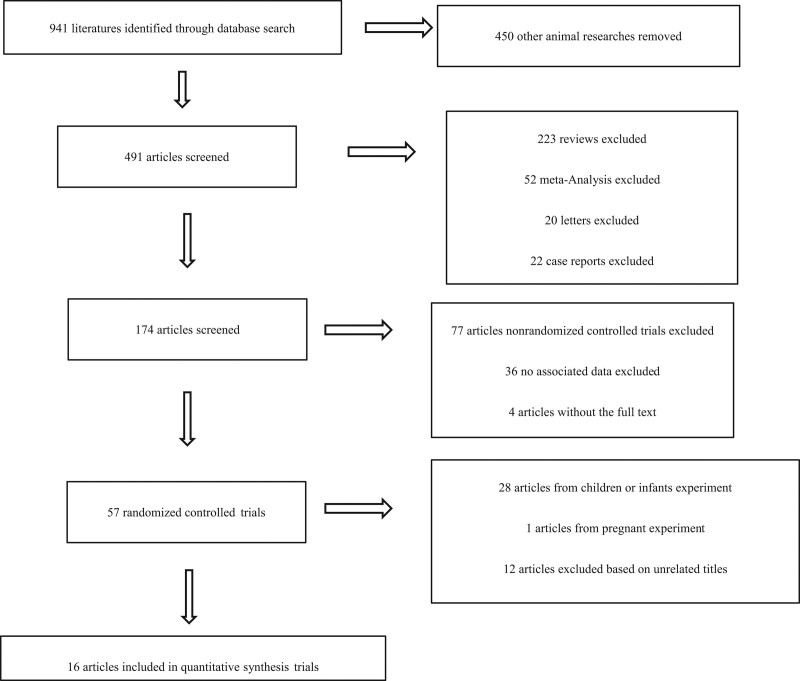
Flow diagram illustrating the literature search and evaluation.

A total of 941 articles were retrieved from the databases. A total of 450 articles from animal experiments, 223 articles from review, 52 articles from meta-analysis, 20 articles from letters, 22 articles from case reports, 4 articles without the full text, 77 articles nonrandomized controlled trials excluded, and 36 articles with incomplete data were excluded, 28 articles from children or infants experiment, 1 articles from pregnant experiment, and 12 articles excluded based on unrelated titles. Thus, a total of remaining 16 publications met the inclusion and exclusion criteria, and details from the trials were extracted separately. Figure 1 shows a flowchart of article selection and inclusion. Because of the heterogeneity of patients, trial methods, and the large variety of outcome measurement used in these trials, pooling of data for meta-analysis was inappropriate. Therefore, the results were summarized qualitatively.

### 3.2. Basic information of the literature

Table 1 presents the basic information of the included studies. In these articles, they used some measures to evaluate BSS, which is defined as follows: separate hard lumps such as nuts; sausage but lumpy; like a sausage or snake but with cracks on its surface; like a sausage or snake, smooth and soft; soft blobs with clear cut edges; fluffy pieces with ragged edges, a mushy stool; and watery, no solid pieces; The IBS symptoms from four aspects: abdominal pain (degree and frequency), bloating, satisfaction with bowel habits, and overall interference with QOL.

**Table 1:** Basic information of the included literature.

Author year	Country and region	T/C	Gender male/female	Trial duration	Pathogeny	Intervention	Outcome measures	Side effects
Sun et al 2018^[28]^	Shandong Province, China	105/95	T:63/42 C:53/42	4 wk	IBS-D	CB	1, 2, 3, 4, 5, 6	a, b, c
Ishaque et al 2018^[29]^	Dhaka, Bangladesh,	181/179	T:136/45 C:145/34	16 wk	IBS-D	Multi-strain probiotic formulation	1, 2, 5, 7	N
Franko et al 2019^[30]^	Des Moines, American	67/68	T:32/35 C: 37/31	30-d	Major abdominal operations	Probiotics	3	N
Skrzydło-Radoman ´ska et al 2020^[31]^	Lublin, Poland	35/33	T:10/25 C: 9/24	8 wk	IBS-D	Synbiotic preparation	1, 2, 7, 8	d, e, f
Hatanaka et al 2018^[32]^	Kanagawa, Japan	40/42	T:24/16 C: 25/17	8 wk	Healthy volunteers	*Bacillus subtilis* C-3102	3, 4, 6, 9	N
Fuke et al 2017^[33]^	Tochigi, Japan	20/22	T:9/11 C: 12/10	12 wk	Healthy subjects	*Lactobacillus brevis* KB290 and b-carotene	1, 10	N
Maity et al 2018^[34]^	Maharashtra, India	30/30	T:20/10 C: 20/10	12 wk	Acute diarrhea	LAB	1	N
Gupta et al 2020^[35]^	Maharashtra, India	20/20	T:13/7 C: 15/5	80 d	Irritable bowel syndrome	*Bacillus coagulans* LBSC	1, 2, 3	N
Gomi et al 2018^[36]^	Tokyo, Japan	39/40	T:19/20 C: 19/21	4 wk	Healthy adults	*Bifidobacterium bifidum* YIT 10347	2, 3, 9	e, f
Han et al 2016^[37]^	Gimpo, Republic of Korea	23/23	T:13/10 C: 11/12	4 wk	Irritable bowel syndrome	Double-coated probiotics	1, 6, 10	N
Zhao et al 2017^[38]^	Sichuan Province, China	40/40	T:24/16 C: 16/24	7 d	Enteral nutrition in gastric cancer patients	Fiber and probiotics	1, 2	N
Soares et al 2017^[39]^	Curitiba, Brazil	29/29	NG	5 d	Malnutrition and antibiotic use	Sporulated *Bacillus* strain	3	N
Hod et al 2017^[40]^	Tel-Aviv, Israel.	54/53	T:0/54 C:0/53	8 wk	IBS-D	Probiotic mixture	1, 2, 3, 6, 11	N
Sharif et al 2017^[41]^	Kashan, Iran	50/50	T:27/23 C: 25/25	5 d	Dysentery	Probiotics	3	N
Barker et al 2017^[42]^	WI, USA	16/15	T:5/11 C:4/11	28 d	*Clostridium difficile* infection	Probiotics	3	N
Hod et al 2018^[43]^	Tel Aviv, Israel	51/46	T:0/51 C:0/46	8 wk	IBS-D	Multispecies probiotic	1, 3, 6, 11	N

Data are expressed as mean ± SD. Outcome measures: 1, abdominal pain; 2, bloating; 3, stool frequency; 4, BSS; 5, IBS-QOL score; 6, Fecal microbiota analysis; 7, The IBS-SSS score; 8, The IBS-GIS score; 9, GSRS; 10: IL-10/IL-1b/IL-12; 11: hs-CRP. a: worse abdominal pain; b: worse bloating; c: hyperactive bowel sound; d: nausea; e: headache; f: rash.

BSS = Bristol stool scale, C = controlled group, CB = *Clostridium butyricum*, GSRS = Gastrointestinal symptom rating scale, IBS-D = diarrhea-dominant irritable bowel syndrome, LAB = lactic acid bacillus, NR = not reported, T = treatment group, SD = standard deviation.

The IBS-QOL^[44]^ score ranges from 0 to 100 points, with higher scores indicating a better QOL. The score contained 34 questions on eight aspects: dysphoria, interference with activity, body image, health worry, food avoidance, social reaction, sexual concern, and relationship. Stool consistency was assessed using the 7-point BSS,^[45]^ with a higher score indicating a softer stool.

The IBS-SSS is a 5-item instrument used to measure the severity of abdominal pain, frequency of abdominal pain (number of days with abdominal pain over the last 10 days), severity of abdominal distension, dissatisfaction with bowel habits, and interference with quality of life, each on a 100-point scale.^[46]^ Thus, the total score can range from 0 to 500 points. IBS severity had the following defined ranges: mild, 75 to 174; moderate, 175 to 300; and severe,  > 300.

IBS-GIS assessed IBS symptoms using a patient-defined 7-point Likert scale ranging from symptoms substantially worse (1 point) to substantially improved (7 points). Patients answered the question, “Have you felt any change in the severity of your symptoms over the past 7 days compared to how you felt before the medicine was taken?” The answers were recorded based on the 7-point scale: 1 point – “I feel that the symptoms have worsened significantly”; 2 points – “I feel that the symptoms have moderately worsened”; 3 points – “I feel that the symptoms have slightly worsened”; 4 points – “I feel no change”; 5 points – “I feel a slight improvement”; 6 points – “I feel moderate improvement”; 7 points – “I feel significant improvement”. The IBS-GIS score indicated an improvement if the score was > 4, a worsening if it was < 4, and no change if it was 4.

Gastrointestinal symptom rating scale symptoms are as follows: “epigastric pain”, “heartburn”, “acid reflux”, “hunger pain”, “nausea”, “rumbling”, “bloating”, “abdominal sounds”, “flatus”, “constipation”, “diarrhea”, “urgent need to have a bowel movement”, and “a feeling of incomplete evacuation”. The severity of each symptom was evaluated on a seven-point scale, with the smaller numbers indicating less severe symptoms. The subscales of acid reflux score, abdominal pain score, dyspepsia score, diarrhea score, and constipation were calculated from the average values of the relevant items. The overall score was calculated as the average of the acid reflux score, abdominal pain score, dyspepsia score, and diarrhea scores.

The intensity of abdominal pain was assessed each day as abdominal pain over the previous 24 h on an 11-point Likert scale ranging from 0 (no pain) to 10 (worst pain imaginable).

## 4. Results of meta-analysis

### 4.1. Stool frequency

**Figure 2.**
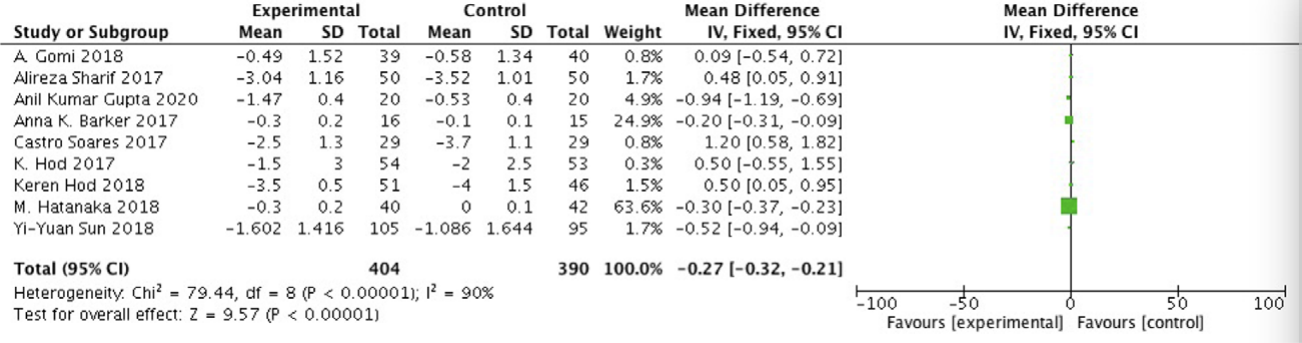
Compared the changes in stool frequency after probiotics intervention between the experimental and control groups, and there was obvious heterogeneity among studies (*P* < .01, *I*^2^ = 90%). The results show that, compared to simple Western medicine treatment or a placebo, the added use of probiotics can improve diarrhea to some extent [WMD = −0.27, 95% CI: −0.32 to −0.21, *P* < .01]. WMD = Weighted mean difference.

A total of nine studies were used stool frequency as an evaluation index and compared the changes in stool frequency after probiotics intervention between the experimental and control groups, and there was obvious heterogeneity among studies (*P* < .01, *I*^2^ = 90%). Therefore, the random effects model was selected for the meta-analysis. The results show that, compared to simple Western medicine treatment or a placebo, the added use of probiotics can improve diarrhea to some extent, Compared with the control group, the difference was statistically significant when compared with the control group (WMD = −0.27, 95% CI: −0.32 to −0.21, *P* < .01) (Fig. 2).

### 4.2. BSS

**Figure 3.**
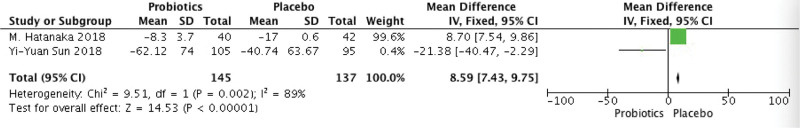
Compared the changes in BSS after probiotics intervention between the two groups, and there was obvious heterogeneity among studies (*P* < .01, *I*^2^ = 89%). Compared to placebo, the difference was statistically significant (WMD = 8.59, 95% CI: 7.43–9.75, *P* < .01). BSS = Bristol stool scale, WMD = Weighted mean difference.

Two of the included studies adopted BSS and then compared the changes in BSS after probiotics intervention between the two groups, and there was obvious heterogeneity among studies (*P* < .01, *I^2^* = 89%). Therefore, the random effects model was selected for the meta-analysis. The results showed that, compared to placebo, the difference was statistically significant (WMD = 8.59, 95% CI: 7.43–9.75, *P* < .01) (Fig. 3).

### 4.3. IBS-QOL score

**Figure 4.**
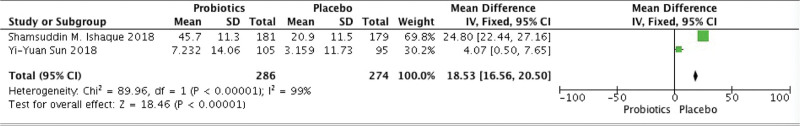
Two studies used the IBS-QOL score as an evaluation indexes, and there was obvious heterogeneity among the studies (*P* < .01, *I*^2^ = 99%). The results showed that, compared to placebo, the difference was statistically significant (WMD = 18.53, 95% CI: 16.56–20.50, *P* < .01). IBS -QOL = Irritable Bowel Syndrome-quality of life, WMD = Weighted mean difference.

Two studies used the IBS-QOL score as an evaluation indexes, and there was obvious heterogeneity among the studies (*P* < .01, *I*^2^ = 99%). Therefore, the random effects model was selected for the meta-analysis. The results showed that, compared to placebo, the difference was statistically significant (WMD = 18.53, 95% CI: 16.56–20.50, *P* < .01) (Fig. 4).

### 4.4. The IBS-SSS score

**Figure 5.**
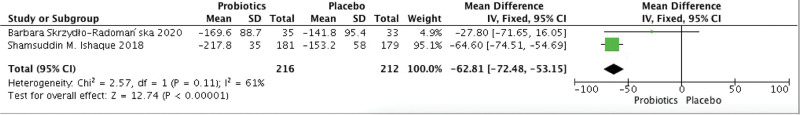
Compared the changes in this index after probiotics intervention between the probiotics group and the placebo group, and there was heterogeneity among studies (*P* = .01, *I*^2^ = 61%). The results showed that, compared to placebo, the difference was statistically significant (WMD = −62.81, 95% CI: −72.48 to −53.15, *P* < .01). WMD = Weighted mean difference.

Two of the included studies adopted the IBS-SSS score, and then compared the changes in this index after probiotics intervention between the probiotics group and the placebo group, and there was heterogeneity among studies (*P* = .01, *I*^2^ = 61%). Therefore, the random effects model was selected for the meta-analysis. The results showed that, compared to placebo, the difference was statistically significant (WMD = −62.81, 95% CI: −72.48 to −53.15, *P* < .01) (Fig. 5).

### 4.5. Fecal microbiota analysis

5 of the included literatures adopted Fecal microbiota analysis (Table 2), In the study of Sun,^[28]^ The fecal microbiota analysis shows a different microbial community after treating and a typical genus, *Clostridium sensu stricto*, is decreased. According to Hatanaka,^[32]^ the relative abundances of two bacterial genera showed significant changes due to ingestion: significantly increased *Lachnospira* and significantly decreased *Actinomyces*; Han^[37]^suggested among the six representative symptoms of IBS, correlation between urgency and clinical parameters was noted. Urgency showed a negative correlation with increased numbers of gut microbiota; In the study of Hod,^[40]^ The fecal microbial analysis demonstrated increased proportions of Bi dbacterium, *Lactobacillus* and *Streptococcus* genera to which the 11 strains of the Bio-25 product belong, at the end of treatment compared with baseline; Hod^[43]^suggested, at 8 weeks of therapy, patients who received the BIO-25 had significantly higher relative proportions of *Lactobacillus* and *Lactococcus*.

**Table 2:** Raw outcomes for change of Intestinal flora through Probiotics by individual trials.

Author year	Intervention	Intestinal flora
Sun et al 2018^[34]^	CB	*Clostridium sensu stricto*↓
Hatanaka et al 2018^[38]^	*Bacillus subtilis* C-3102	*Lachnospira*↑
Han et al 2016^[43]^	Double-coated probiotics	Actinomyces↓↓
		Bifidobacterium↑
Hod et al 2017^[47]^	Probiotic mixture	Bi dbacterium↑
Hod et al 2018^[48]^	Multispecies probiotic	*Lactobacillus* and *Streptococcus* genera↓
		*Lactobacillus acidophilus*↑

CB = *Clostridium butyricum*.

### 4.6. Side effects

Probiotics are generally considered generally safe in immunocompetent patients, and undesired side effects of probiotics are rare, although possibly under-reported.

## 5. Discussion

Probiotics are widely used in the treatment of IBS and have been shown to be modestly effective. Probiotics are live microorganisms that, when administered in adequate amounts, confer a health benefit on the host’.^[49]^ Synbiotics are a mixture of probiotics and prebiotics that act synergistically to promote the growth and survival of beneficial microorganisms in the gut.^[47]^ The rationale for the use of probiotics in the management of IBS is their potential to correct dysbiosis or to stabilize the host microbiota. A decreased abundance of *Bifidobacterium* and *Lactobacillus* species,^[50]^ and an increase in *Gammaproteobacteria* species^[51]^are frequently reported in IBS studies. Furthermore, PCR-denaturing gradient gel electrophoresis analysis of fecal samples from IBS patients revealed greater temporal instability of the microbiota compared with healthy controls.^[52]^ There is accumulating evidence showing that certain probiotics may be capable of significantly reducing abdominal pain, abdominal distension and flatulence while, at the same time, increasing health-related QOL in IBS patients.^[48]^

Numerous studies have described the mechanism of IBS onset as a shift from “normal and healthy” gut to “dysbiosis and unhealthy” gut, where the gut microbial community plays a pivotal role.^[48,53–55]^ Healthy microbiome-modulated intestinal homeostasis is therefore a fundamental therapeutic paradigm in which probiotics could offer a promising healthcare solution for IBS.^[56]^ Probiotics are live bio-therapeutics^[57]^ that offer a promising route for treating various gastrointestinal ailments such as diarrhea, indigestion, nutrient malabsorption, inflammatory bowel disease, ulcerative colitis, and Crohn disease, without the risk of spreading antibiotic resistance in microorganisms. The use of probiotics in the treatment of IBS has been reported to be strongly effective in several trials.^[58]^ These studies have suggested the beneficial effects of probiotics by way of improving the immune response, enhancing intestinal permeability, and altering colonic fermentation.^[59,60]^ Emerging studies have supported the use of probiotics in the treatment of IBS. Probiotics, as drugs for the treatment of a variety of diseases, have opened up new avenues for the treatment of diarrhea, making clinicians have a choice when using drugs.

However, in the process of our meta-analysis, we have to acknowledge some limitations of the study. First, it was found that there are still many places worthy of deliberation in the existing trials. Besides, the vast majority of our studies originated from eastern countries; thus, extrapolation of these results to Western populations is questionable. Moreover, the indicators used in these experiments to describe the stool situation are lack of unified and recognized indicators, which makes our analysis work more difficult. Results of the meta-analysis showed that probiotics have therapeutic effects on diarrhea caused by various pathogens. Therefore, the indications of the drug still need to be further improved through large-scale clinical trials. The age, gender, pathogeny of disease, regimens, doses, duration, center settings, population and other aspects of the included cases are greatly affected by the trial implementers, and there is selective bias. The effect in some occasions was assessed by few studies; thus, the evidence to support may be low.

Besides, some studies did not select the most clinically significant index to evaluate the curative effect, and the follow-up time was short. There are few articles on prognostic indicators such as recurrence rate. The effectiveness and safety of probiotics in patients with diarrhea still need to be further evaluated. In some trials, placebo was not used in the control group, and the blind method was not applied to the trial implementers and subjects. There is the possibility of inducing patients’ subjective responses, and the implementation deviation cannot be ruled out; most of the included studies had small samples, and the positive results accounted for the vast majority. There is a possibility of publication deviation. In short, the disadvantages of the existing design scheme affect the repeatability of the test and the credibility of the conclusion. At present, there are few articles on the action mechanism of action of the drug, and most have been published in animal experiments. Therefore, the efficacy of probiotics on diarrhea still needs to be verified by more sample, high-quality, and multi-center clinical trials, so as to guide the clinic in the future.

## 6. Conclusion

In conclusion, it is evident from the presented articles that the probiotics are safe and effective for improving the diarrhea and related discomfort, as evaluated through these human clinical trials. This implies that probiotic supplements may be candidates for the treatment of diarrhea. Moreover, to some extent, probiotic supplements showed a satisfactory effect on the improvement of the stool morphology and IBS symptoms in the stool of diarrhea-predominant IBS patients. This may be due to a shift of beneficial intestinal microbiota.

## Author contributions

**Conceptualization:** Xianghua Ma.

**Data curation:** Fujie Wang, Xianghua Ma.

**Formal analysis:** Fujie Wang.

**Supervision:** Xianghua Ma.

**Writing – review & editing:** Ting Zhao, Weiwei Wang, Qianqian Dai.
